# Validity of resting heart rate derived from contact-based smartphone photoplethysmography compared with electrocardiography: a scoping review and checklist for optimal acquisition and reporting

**DOI:** 10.3389/fdgth.2024.1326511

**Published:** 2024-02-29

**Authors:** James D. Mather, Lawrence D. Hayes, Jacqueline L. Mair, Nicholas F. Sculthorpe

**Affiliations:** ^1^ Sport and Physical Activity Research Institute, School of Health and Life Sciences, University of the West of Scotland, Glasgow, United Kingdom; ^2^ Future Health Technologies, Singapore-ETH Centre, Campus for Research Excellence and Technological Enterprise (CREATE), Singapore, Singapore; ^3^ Saw Swee Hock School of Public Health, National University of Singapore, Singapore, Singapore

**Keywords:** photoplethysmography, PPG, mobile, heart rate, validity

## Abstract

**Background:**

With the rise of smartphone ownership and increasing evidence to support the suitability of smartphone usage in healthcare, the light source and smartphone camera could be utilized to perform photoplethysmography (PPG) for the assessment of vital signs, such as heart rate (HR). However, until rigorous validity assessment has been conducted, PPG will have limited use in clinical settings.

**Objective:**

We aimed to conduct a scoping review assessing the validity of resting heart rate (RHR) acquisition from PPG utilizing contact-based smartphone devices. Our four specific objectives of this scoping review were to (1) conduct a systematic search of the published literature concerning contact-based smartphone device-derived PPG, (2) map study characteristics and methodologies, (3) identify if methodological and technological advancements have been made, and (4) provide recommendations for the advancement of the investigative area.

**Methods:**

ScienceDirect, PubMed and SPORTDiscus were searched for relevant studies between January 1st, 2007, and November 6th, 2022. Filters were applied to ensure only literature written in English were included. Reference lists of included studies were manually searched for additional eligible studies.

**Results:**

In total 10 articles were included. Articles varied in terms of methodology including study characteristics, index measurement characteristics, criterion measurement characteristics, and experimental procedure. Additionally, there were variations in reporting details including primary outcome measure and measure of validity. However, all studies reached the same conclusion, with agreement ranging between good to very strong and correlations ranging from *r *= .98 to 1.

**Conclusions:**

Smartphone applications measuring RHR derived from contact-based smartphone PPG appear to agree with gold standard electrocardiography (ECG) in healthy subjects. However, agreement was established under highly controlled conditions. Future research could investigate their validity and consider effective approaches that transfer these methods from laboratory conditions into the “real-world”, in both healthy and clinical populations.

## Introduction

### Rationale

Photoplethysmography (PPG) can provide important clinical outcome measures and has been used for the diagnosis, monitoring, and screening of various diseases and disorders (1). “Photoplethysmography” consists of “photo,” meaning light; “plethysmo,” meaning volume; and “graphy” meaning recording (2). PPG was first suggested as a technique for measuring blood volume changes by Hertzman in 1937 (3, 4). PPG is a measurement of light either absorbed (transmissive photoplethysmography) or reflected (reflective photoplethysmography) by human tissues (1), and is based on optical properties such as absorption, scattering and transmission (5). Transmissive PPG measures light that passes through the various human tissues and is mainly used at the distal parts of the body where those tissues are thin, for example at the fingers, toes, and earlobes. Reflective PPG measures scattered light that irradiates skin tissue and produces a reduced light intensity (6). While transmissive PPG exhibits more stable PPG performance (7) since the reflective type of signal is degraded, the latter has the advantage of a greater number of measurement sites such as the forehead, wrist, carotid artery, and esophagus, where transmissive PPG would be difficult (8, 9).

As such, PPG data is explained by Beer-Lambert's Law which defines resultant light intensity by the extinction coefficient, concentration, and optical path length of a medium when light passes through it (10). The Beer–Lambert law can be described by:

$$I=I_{0}\cdot e^{-\varepsilon (\lambda )\;\cdot \rho \cdot d}$$

Whereby: the transmitted light intensity (I) through a medium will decrease exponentially in irradiated light intensity (*I*_0_) in relation to the absorption coefficient (*ε*), where (*λ*) is the specific absorptivity, characteristic of the traversed tissue and dependent on the light wavelength *λ*, *ρ* is the density of the tissue, and d is the light pathlength (6).

Since most of these factors are constant for a given tissue the signal quality is mainly impacted through the later part of the equation, through manipulation of *λ*, *ρ* and *d*, which can be modified through measurement site selection, wavelength selection and contact pressure, resulting in a reduced *ε*, which could explain why fingers and earlobes are preferred.

Various PPG devices have been utilized in clinical practice (1). However, since the release of the first iPhone in 2007, smartphones have been widely adopted globally (11) and are now considered a tool with high utility, avoiding some major pitfalls of traditional data collection techniques. The traditional approach where an individual's health is monitored periodically, often by appointment, may not be an accurate representation of the possible variations in physiological measurements that occur longitudinally (12, 13). Moreover, smartphone technology and embedded cameras allow PPG acquisition without the need for additional, potentially costly, external devices (14) and could be suitable for targeting populations in traditionally underserved groups (15) particularly those whose demographic, geographic, or economic characteristics negatively affect health care access and delivery (16, 17). Therefore, telemedicine technologies are becoming more widely adopted in practice, especially since the recent COVID-19 pandemic, which highlighted the need for vital signs evaluated using telemonitoring (14, 18, 19). As a result, the proliferation of smartphone-based telemedicine appears to be here to stay and could address the United Nations Sustainable Development Goals (UN SDGs) (20), in particular UN SDG 3 (21).

Smartphone PPG has been previously utilized to estimate resting heart rate (RHR) through the measurement of distal pulse rate (PR) at rest, during exercise, and whilst completing mental tasks (1). However, at the time of writing, there is no consensus on what metric should be used to establish the validity of smartphone-based PPG or under what conditions. Another issue is that to convert the PPG signal, a mathematical algorithm is required which not only affects smartphone performance but also validity and reliability. This is problematic given the proliferation of telemedicine, and it is therefore essential mobile health (mHealth) technologies are considered reliable and valid compared to gold standard measurements before universal adoption (22). In this context, De Ridder et al. (23) conducted a meta-analysis of articles published between 1st January 2009 and 7th December 2016 investigating the use of smartphones to measure PR by performing PPG in comparison with a range of methods, including ECG, pulse oximetry and radial pulse. Although these methods suffer various pitfalls, comparisons with multiple validation methods could strengthen smartphone device and application validity. Results revealed good agreement between smartphone-derived (HR-PPG) and validated method-derived RHR. These authors therefore concluded that RHR obtained from a smartphone PPG signal could be used as an alternative to traditional methods, such as ECG, in an adult population, in the right context. However, De Ridder et al. (23) highlighted several limitations to the included studies. Firstly, there was high statistical heterogeneity between studies, ostensibly due to participant characteristics, measurement conditions, and the smartphone devices utilized (23). Secondly, the latest IOS device reviewed was the iPhone 5 (released 2012) and the latest android was the Samsung Galaxy S4 (released 2013). Emerging evidence suggests advancements in technology, such as the availability of various camera positions (i.e., front-facing vs. rear-facing) and the advent of multiple lenses, could result in improvements in PPG acquisition (14).

These technological enhancements are promising for the telemedicine sphere as HR-PPG could be considered a population-level biomarker, utilized for screening, surveillance, and to monitor responses to policy interventions in epidemiology and public health. Population-level biomarkers are easy to measure in the real-world, low-cost and scalable (24). RHR has considerable population-level applicability and can predict adverse outcomes and the development of disease. As smartphone ownership is increasing [80% of over 65-year-olds own a smartphone in the UK (25, 26)], and smartphone HR-PPG removes the barrier to scalability of “wearable” ownership, valid contact-based HR-PPG from a smartphone device has significant scope for public health surveillance. However, before that goal is reached, it is imperative to consider the existing literature in terms of HR-PPG validity.

Two approaches of measuring PR via PPG are known: contact and non-contact. With contact PPG, PR is measured by placing a finger on the phone rear camera, while in non-contact, imaging photoplethysmography (iPPG) is extracted from the face, without the need for direct skin contact. iPGG has some advantages over contact-based PPG, such as detecting PR in crowds and at long-distance (27, 28). However, in general, contact PPG exhibits better accuracy than non-contact PPG (29). Considering that contact-based PPG is generally more accurate than non-contact PPG, and our group's interest in this methodology, we were interested in the validity of RHR acquisition from PPG utilizing contact-based smartphone devices.

### Objectives

As a result of the importance of using validated PPG for telemedicine, and the rapidly improving technology, we aimed to conduct a scoping review assessing the validity of RHR acquisition from PPG (referred to as HR-PPG) utilizing contact-based smartphone devices against gold standard ECG (referred to as HR-ECG). Our four specific objectives of this scoping review were to 1) conduct a systematic search of the published literature concerning contact-based smartphone device-derived PPG, 2) map study characteristics and methodologies, 3) identify if methodological and technological advancements have been made, and 4) provide recommendations for the advancement of the investigative area.

## Methods

### Protocol and registration

The review was not preregistered, as scoping reviews are not. This review was conducted and reported in accordance with the Preferred reporting items for systematic reviews and meta-analyses extension for scoping reviews (PRISMA-ScR) guidelines (30).

### Eligibility criteria

Studies were included if the measurement of HR-PPG was conducted via the front or rear facing camera of a smartphone by contact-based PPG. Only studies compared with the gold standard measurement [electrocardiography (ECG)], were included. Studies were excluded if the index measurement was conducted with a device connected to a smartphone, such as a mobile sensor, medical device or wearable device; the paper did not include validity assessment of HR-PPG and HR-ECG as an outcome measurement; the study used a clinical population (we assumed healthy population unless stated otherwise); the paper was not an original article (i.e., utilized a database from a secondary source); the paper was a review; there was no abstract or full text available.

### Literature search

We conducted a systematic literature search of ScienceDirect, PubMed and SPORTDiscus from January 1st, 2007, to November 6th, 2022, with the following search key: (((((“validity”) AND (“mobile”)) AND (“photoplethysmography”)) OR (“PPG”)) AND (“heart rate”)) NOT (“wearable”) AND [2007:2022(pdat)], which were developed through examination of previously published original and review articles. Filters were applied to ensure only literature written in English were included. Reference lists of included studies were manually searched for additional eligible studies.

### Study selection

Studies were identified by the first author and evaluated by JDM and LDH independently and compared in an unblinded and standardized manner. Once database searches were complete, all studies were downloaded to a single reference list [utilizing Zotero software (version 6.0.26)] and duplicates were removed. First, titles and abstracts were screened for eligibility (JDM). Full text articles were then read and coded in relation to exclusion criteria, utilizing “tags” in Zotero [version 6.0.26], which was reviewed by the second author (LDH). This process involved a thorough assessment of all eligibility criteria with authors JDM and LDH confirming inclusion and exclusion. Additionally, disagreements were addressed by a third reviewer (NFS).

### Data extraction

Data extracted from each study included author(s), sample size, participant sex, country of study, age, skin pigmentation, if participants were considered healthy, smartphone model, name of application utilized, whether the application was commercially available, index measurement sampling rate, camera position and resolution, flash (torch) settings, channel used for computations, ECG device utilized, electrode placement, ECG processing information, instructions given to participants, dietary control, participant posture, region of interest, breathing pattern, environmental conditions, stabilization period, duration of measurement, number of attempts or trials, primary outcome measures and measures of validity.

### Outcome measures

Our primary interests were measurements of validity and mean differences between heart rate via gold standard ECG measurement (HR-ECG), and pulse rate measured by contact-based smartphone PPG (HR-PPG). Additionally, issues that arose regarding the reporting and conducting of HR-PPG validity assessment were compiled into a checklist (Table 1).

**Table 1:** Items to consider when reporting validity protocols for the acquisition of RHR via contact-based PPG, using smartphone devices.

Domain	Item	Description	Tick
Target population	1	BMI (kg/m^2^)	Ο
	2	Body height (m or cm)	Ο
	3	Skin tone (State scale utilized and distribution)	Ο
	4	Sample size (number of subjects)	Ο
	5	Participant age (years)	Ο
	6	Participant sex (*n* = male, *n* = female)	Ο
	7	Healthy vs. clinical [if clinical report condition and medication(s)]	Ο
	8	Sampling method (random, convenient etc.)	Ο
Criterion measure	9	ECG manufacturer's details (model and brand)	Ο
	10	Number of leads	Ο
	11	Number of channels	Ο
	12	Pre-measurement preparation (i.e., skin preparation procedure)	Ο
	13	Placement according to manufacturer's details or state if otherwise	Ο
	14	ECG sampling frequency (Hz)	Ο
	15	Type of electrode (make, wet vs. dry)	Ο
Index measure	16	Device manufacturer's details	Ο
	17	Application name, version and commercial availability	Ο
	18	Sampling rate (Hz)	Ο
	19	Camera(s) utilized (i.e., front- and/or rear-facing)	Ο
	20	Camera resolution (pixels)	Ο
	21	Torch/flash setting during measurements	Ο
	22	Wavelength channel used for computations (RGB)	Ο
Testing conditions	23	Clear and concise participant instructions	Ο
	24	State dietary control (duration, hours prior to testing)	Ο
	25	Report medication (including dosage)	Ο
	26	Any physical exercise restrictions imposed (report in hours prior)	Ο
	27	Participant posture(s)	Ο
	28	Body region(s) measured	Ο
	29	Breathing pattern (spontaneous vs. metronome rate)	Ο
	30	Environmental conditions (Environmental noise, temperature, ambient lighting conditions, indoors, outdoors, laboratory vs. free-living environment)	Ο
	31	Stabilization period (minutes or seconds)	Ο
	32	Duration of measurement (minutes or seconds)	Ο
	33	Number of attempts	Ο
	34	Artificially induced motion artifact (MA) should describe the method used to induce the MA (i.e., shaking the device) and the frequency of the MA induced (Hz)	Ο
	35	Define mental stress tasks (if any) (i.e., mental arithmetic/mirror tracing)	Ο
	36	State if criterion and index measurements were simultaneous	Ο
	37	Testing conditions reviewed with expert input (i.e., physiologist)	Ο
Data processing	38	State PPG noise removal (motion artifact, baseline wandering and hypoperfusion) technique(s) (i.e., frequency domain filter, high-pass filter etc.)	Ο
	39	State cut off frequencies for noise removal.	Ο
	40	State pre-processing techniques (frequency filtering, empirical mode decomposition, wavelet transform etc.).	Ο
	41	State method of peak detection (zero-crossing, local maxima or minima, adaptive threshold, machine learning etc.).	Ο
	42	Report any PPG waveform reconstruction.	Ο
Statistical analysis	43	Report correlation coefficient results utilizing guidelines proposed by Vincent (1999) or justify otherwise.	Ο
	44	Report Post-hoc comparisons utilized.	Ο
	45	Utilize inferential statistics for sample sizes >30 participants.	Ο

## Results

### Study selection

**Figure 1:**
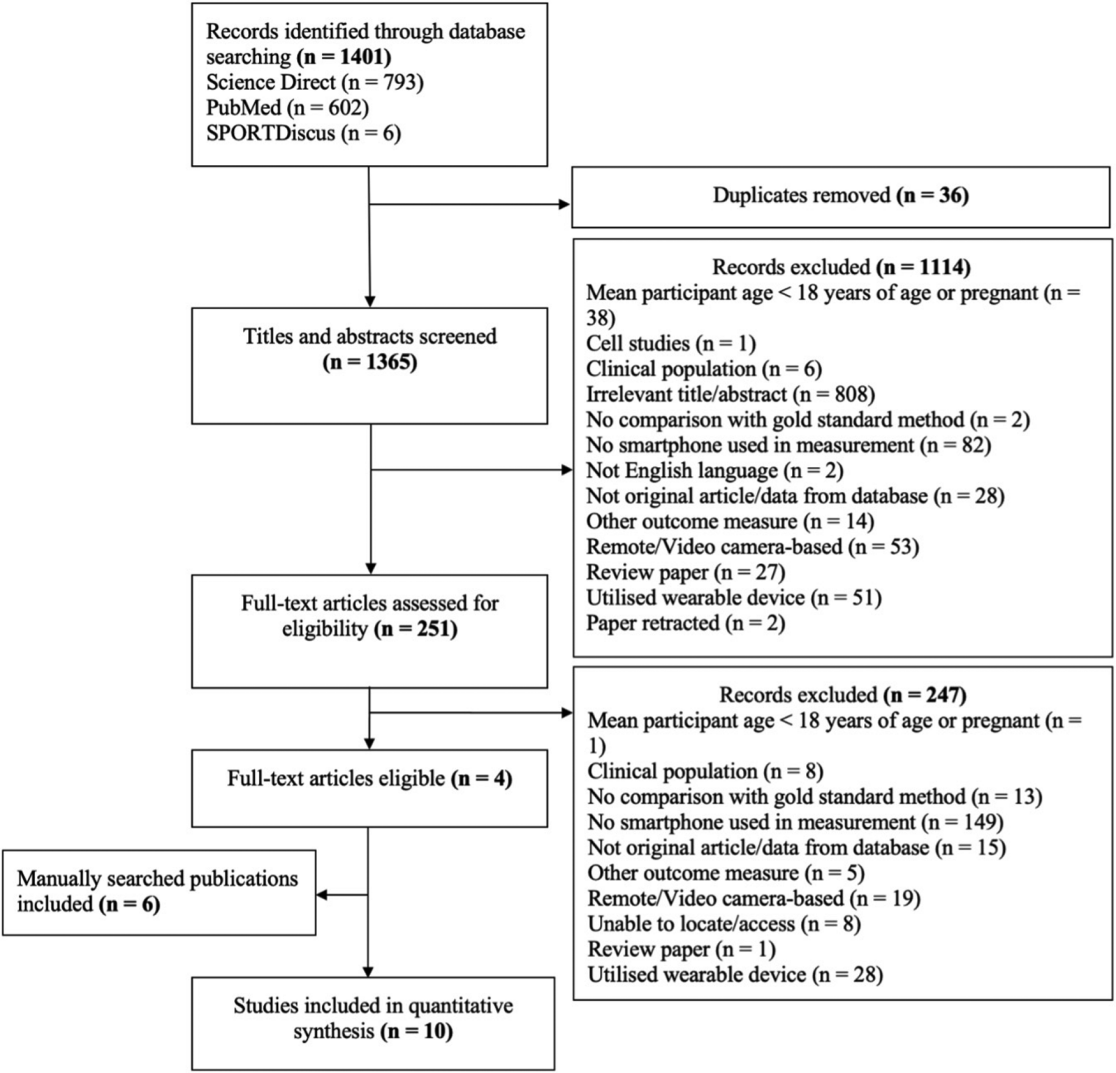
Records identified through reference list searching.

Following initial database searches, 1,401 articles were identified, and 1,365 titles and abstracts were screened once duplicates (*n* = 36) were removed. These were screened for inclusion, resulting in 251 full text articles being screened. Of these 247 were excluded and four remained. A further six articles were manually identified by consulting reference lists of the included four articles, resulting in a further six articles, and therefore a total of 10 articles were included in analysis (Figure 1).

### Study characteristics

Of the ten studies included in the review, all (100%) reported the country of study, which were upper-middle to high income countries. Sample sizes were reported in all studies (100%) (11, 31–39) and ranged from one to 50 participants. Seven (70%) reported the number of male and female participants, of which most were male, and seven (70%) studies reported participant age (33–39). Only one study (10%) reported participant skin colour (36) and five (50%) reported participants’ health status (11, 34–37) (Table 2).

**Table 2:** General study information of investigations concerning smartphone rear-facing PPG measurement and ECG for the determination of heart rate (pulse rate) and descriptive statistics of participants.

Reference	Sample size	Sex (M/F)	Country of study	Participant age (mean ± SD or range)	Skin color	Healthy population
Bánhalmi et al., (37)	50	39/11	Hungary	27	Not reported	Y
Bolkhovsky, Scully and Chon (31)	22	Not reported	United States	Not reported	Not reported	Not reported
Drijkoningen et al., (11)	28	Not reported	Belgium	Not reported	Not reported	Y
Matsumura and Yamakoshi (33)	12	7/5	Japan	21–24	Not reported	Not reported
Matsumura et al.*,* (34)	12	12/0	Japan	20.6 ± 0.76	Not reported	Y
Nam et al.*,* (35)	11	9/2	Korea, United States and China	20–40	Not reported	Y
Nemcova et al.*,* (38)	22	9/13	Czech Republic	18–78	Not reported	Not reported
Nemcova et al.*,* (39)	12	6/6	Czech Republic	21–61	Not reported	Not reported
Scully et al.*,* (32)	1	Not reported	United States	Not reported	Not reported	Not reported
Yan et al.*,* (36)	40	20/20	China	24.7 ± 5.2	von Luschan skin color, median [IQR (Interquartile range)] Male: 23.5 (22–24), Female: 19 (18–25.75), All: 23 (19–25), *P *= .19.	Y

### Index measurement characteristics

**Figure 2:**
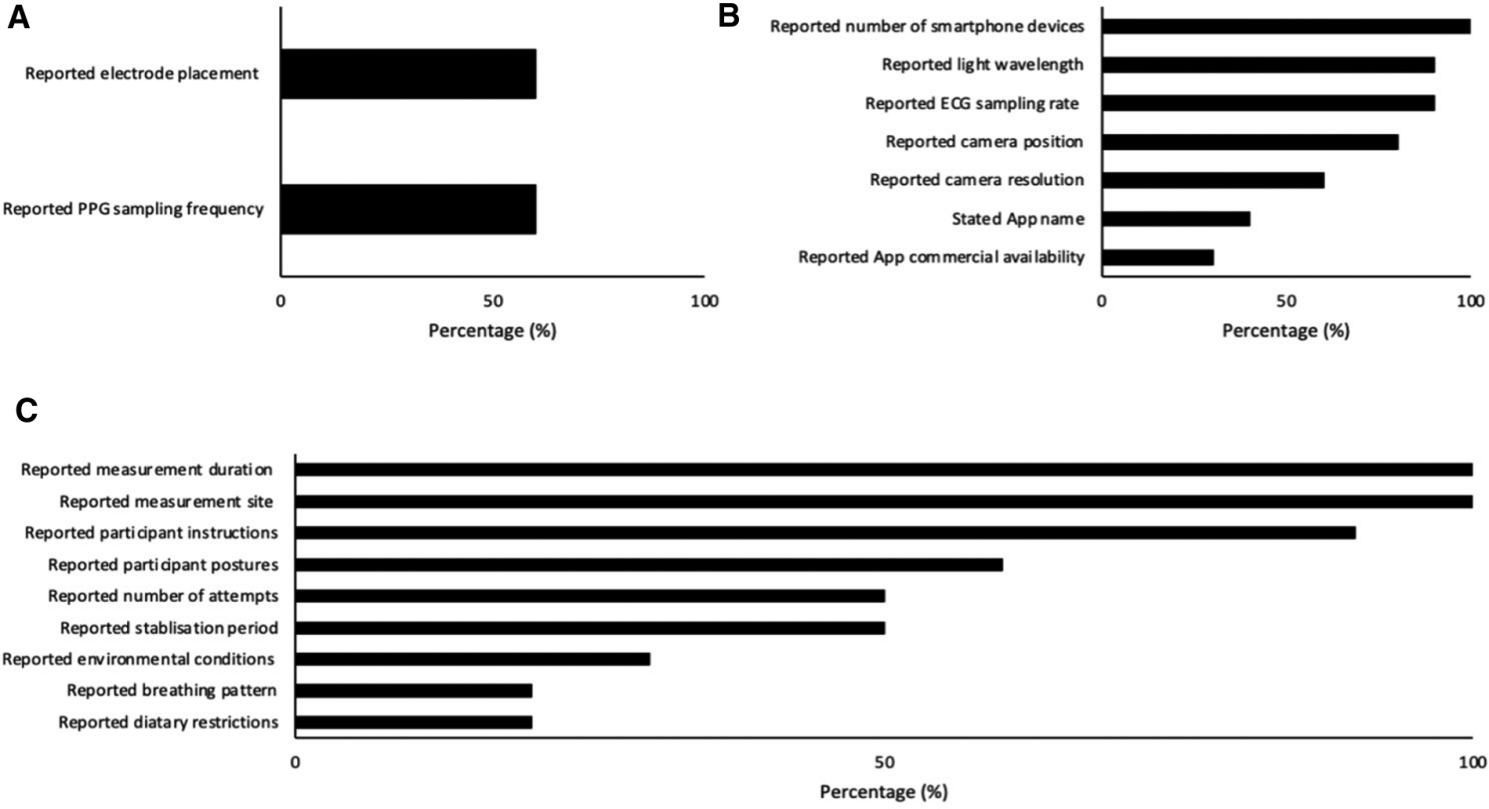
Number of papers reporting index (**A**), criterion (**B**) and environmental (**C**) characteristics.

Index measurement characteristics are displayed in Figure 2A and Table 3 (measurement settings) and Table 4 (device hardware specifications). Eight studies (80%) used a single smartphone for data collection (11, 32–37, 39) and two studies (20%) utilized two or more devices (31, 38). Four articles (40%) stated the name of the smartphone application (33, 34, 36, 38), three (30%) of which were commercially available (33, 34, 38). In the remaining seven studies (70%), commercial availability was not reported (11, 31, 32, 35–37, 39). No studies (0%) reported beat detection algorithm. Eight studies (80%) reported which camera recorded smartphone PPG measurements (32–39) of which the rear-facing camera was utilized for all with torch (flash) turned on. Two studies (20%) failed to report camera location and torch (flash) settings (11, 31). Camera resolution was reported in six studies (60%) (32–35, 37, 39) and varied in resolution. In the remaining studies resolution was not reported (40%) (11, 31, 36, 38). Smartphone sampling rate was reported in nine studies (90%) (11, 31–35, 37–39) and one study (10%) did not report sampling rate (36). Of the nine studies that did report sampling rate six (60%) recorded at 30 Hz (11, 33–35, 38, 39) one (10%) recorded at 30 Hz and 20 Hz, which was dependent on smartphone device (31), one study (10%) recorded at 24.99 Hz (32) and the final study (10%) that reported sampling rate recorded in “slow-motion” capture mode at 240 Hz (37). Nine studies (90%) reported the color channel used during analysis (11, 31–35, 37–39), and in one study (10%) it was not reported (36). Of these studies two studies (20%) utilized red, green and blue color channels (34, 38). Three studies (30%) utilized green only (32, 33, 35). One study (10%) utilized red and green depending on smartphone used (31). Two studies (20%) utilized red only (37, 39). Finally, one study (10%) converted to a single grey value (11).

**Table 3:** Index measurement (smartphone) descriptive statistics for included studies.

Reference	Smartphone model(s) utilized	Name of mobile application	Commercially available (Y/N)	Sampling rate	Camera(s) utilized and resolution	Torch/Flash during measurement (Y/N)	Channel used for computations	Peak detection method
Bánhalmi et al.*,* (37)	iPhone 6	Not reported	Not reported	240 fps (Hz) (“Slow-motion” capture mode)	Rear-facing (720 pixels)	Y [Flash (torch) was set to “maximum”]	Red	Identifying maxima points of the PPG signal
Bolkhovsky, Scully and Chon (31)	iPhone 4S (*n* = 9) and Motorola Droid (*n* = 13)	Not reported	Not reported	iPhone: 30 fps (Hz), Motorola Droid: 20 fps (Hz)	Not reported	Not reported	iPhone (green band) and Motorola Droid (red band)	Identifying maxima points of the PPG signal
Drijkoningen et al.*,* (11)	Samsung Galaxy S4	Not reported	Not reported	30 fps (Hz)	Not reported	Not reported	Converted to a single grey value.	Identifying maxima points of the PPG signal
Matsumura and Yamakoshi (33)	iPhone 4S	iPhysioMeter	Y	30 fps (Hz)	Rear-facing (192 × 144 pixels)	Y	Green	Identifying maxima and minima points of the PPG signal
Matsumura et al.*,* (34)	iPhone 4S	iPhysioMeter	Y	30 fps (Hz)	Rear-facing (192 × 144 pixels)	Y	Red, green, and blue	Identifying maxima and minima points of the PPG signal combined with the detection of peaks using thresholds
Nam et al.*,* (35)	HTC One M8	Not reported	Not reported	30 fps (Hz) (effective frame rate 20–25 Hz)	Rear-facing (176 × 144 pixels)	Y	Green (region closest to the flash of 176 × 72)	Power spectral analysis over a 120 s sample.
Nemcova et al.*,* (38)	(12 smartphone models) The Lenovo Vibe S1 was utilized alongside the participants personal smartphone device. Other smartphones utilized include Honor 7 Lite, Apple iPhone SE, Lenovo S60, Xiaomi Redmi 3, Samsung Galaxy S4, Samsung Galaxy J5, Lenovo Vibeshot, Lenovo S750, Huawei P10, Samsung Galaxy A3 and Apple iPhone 6S.	BP Data Acquisition App	Y	30 fps (Hz)	Rear-facing (Not reported)	Y	Red, green, and blue	Identifying maxima points of the PPG signal
Nemcova et al.*,* (39)	Xiaomi Mi9	Not reported	Not reported	30 fps (Hz)	Rear-facing (720 × 1,280 pixels)	Y	Red	Identifying maxima and minima points of the PPG signal
Scully et al.*,* (32)	Motorola Droid R	Not reported	Not reported	24.99 fps (Hz)	Rear-facing (720 × 480 pixels)	Y	Green (50 × 50-pixel average of a region)	Identifying maxima and minima points of the PPG signal combined with the detection of peaks using thresholds
Yan et al.*,* (36)	iPhone 6S	Cardiio	Not reported	Not reported	Rear-facing (Not reported)	Y	Not reported	Proprietary commercially available app (Cardiio app).

fps, frames per second; Hz, hertz.

**Table 4:** Index device hardware technical specifications for included studies.

Reference	Model	Display	Chip	Camera	Video Recording
Bánhalmi et al.*,* (37)	iPhone 6	Retina HD display	A8 chip with 64-bit architecture	8-megapixel iSight camera with 1.5 µ pixels	1080 p HD video recording (30 fps or 60 fps)
		4.7-inch (diagonal) LED-backlit widescreen Multi-Touch display with IPS technology			
					True tone flash
				Autofocus with Focus Pixels	Slo-mo video (120 fps or 240 fps)
				ƒ/2.2 aperture	Time-lapse video with stabilization
				Optical image stabilization (iPhone 6 Plus only)	Cinematic video stabilization
					Continuous autofocus video
					Take still photos while recording video
			M8 motion coprocessor		
					Improved face detection
				True tone flash	3x zoom
				Five-element lens	Video geotagging
		1,334-by-750-pixel resolution at 326 ppi		Hybrid IR filter	
		1,400:1 contrast ratio (typical)		Backside illumination sensor	
		500 cd/m2 max brightness (typical)		Sapphire crystal lens cover	
		Full sRGB standard		Auto image stabilization	
		Dual-domain pixels for wide viewing angles		Auto HDR for photos	
				Face detection	
				Exposure control	
				Panorama (up to 43 megapixels)	
				Burst mode	
				Tap to focus	
		Fingerprint-resistant oleophobic coating on front		Photo geotagging	
				Timer mode	
		Support for display of multiple languages and characters simultaneously			
		Display zoom			
		Reachability			
Bolkhovsky, Scully and Chon (31)	iPhone 4S	Retina display		8-megapixel camera	
		3.5-inch (diagonal) widescreen Multi-Touch display		Autofocus	
				Tap to focus	
				Face detection in still images	
				LED flash	
		960-by-640-pixel resolution at 326 ppi		Video recording, HD (1080 p) up to 30 frames per second with audio	
		800:1 contrast ratio (typical)			
		500 cd/m^2^ max brightness (typical)			
		Fingerprint-resistant oleophobic coating on front and back		Video stabilization	
				Front camera with VGA-quality photos and video at up to 30 frames per second	
		Support for display of multiple languages and characters simultaneously			
				Photo and video geotagging	
Bolkhovsky, Scully and Chon (31)	Motorola Droid	Information not available	Information not available	Information not available	Information not available
Drijkoningen et al.*,* (11)	Samsung Galaxy S4	Technology	CPU Type	Camera Resolution (Rear)	Video Codec
		FHD sAMOLED	Quad	CMOS, 13MP Camera Resolution (Front)	MPEG4, H.263, H.264, DivX, DivX3.11, VC-1, VP8, WMV7 / 8, Sorenson Spark, HEVC
		Colour Depth	CPU Speed		
		16M	1.9GHz	CMOS, 2MP Flash	
		Size		Power LED (1EA) Auto Focus Yes	Video Resolution
		5″			Full HD (1080 p) Video Playback
		Resolution			Video Frame rate
		1,920 × 1,080			30fps
					Audio Codec
					MP3, AAC, AAC+, eAAC+, AMR-NB / WB, OGG, FLAC, AC-3, apt-X (Bluetooth)
Matsumura and Yamakoshi (33)	iPhone 4S	Retina display		8-megapixel camera	Information not available
		3.5-inch (diagonal) widescreen Multi-Touch display		Autofocus	
				Tap to focus	
		960-by-640-pixel resolution at 326 ppi		Face detection in still images	
		800:1 contrast ratio (typical)		LED flash	
		500 cd/m^2^ max brightness (typical)		Video recording, HD (1080 p) up to 30 frames per second with audio	
		Fingerprint-resistant oleophobic coating on front and back			
				Video stabilization	
				Front camera with VGA-quality photos and video at up to 30 frames per second	
		Support for display of multiple languages and characters simultaneously			
				Photo and video geotagging	
Matsumura et al.*,* (34)	iPhone 4S	Retina display		8-megapixel camera	Information not available
		3.5-inch (diagonal) widescreen Multi-Touch display		Autofocus	
				Tap to focus	
				Face detection in still images	
		960-by-640-pixel resolution at 326 ppi			
				LED flash	
				Video recording, HD (1080 p) up to 30 frames per second with audio	
		800:1 contrast ratio (typical)			
		500 cd/m^2^ max brightness (typical)			
				Video stabilization	
				Front camera with VGA-quality photos and video at up to 30 frames per second	
		Fingerprint-resistant oleophobic coating on front and back			
				Photo and video geotagging	
		Support for display of multiple languages and characters simultaneously			
Nam et al.*,* (35)	HTC One M8	5.0 inch	Qualcomm® Snapdragon™ 801, quad-core CPUs	Primary camera:	Information not available
		Full HD 1080 p		HTC UltraPixel™ camera	
				BSI sensor	
				pixel size 2.0 um	
				sensor size 1/3"	
				ƒ/2.0	
				28 mm lens	
				HTC ImageChip 2.	
				1080 p Full HD video recording with HDR video	
				Secondary camera:	
				Capture depth information	
Nemcova et al.*,* (38)	Lenovo Vibe S1	Capacitive touchscreen,	MT6752 64-bit 1.7 GHz Octa-Core	Rear:	Information not available
		16M colors, 5-point multitouch 5.0" (1,920 × 1,080) Full HD		13MP AF with dual-color flash,	
				PDAF, BSI sensor	
		IPS display @ 440 ppi			
	Honor 7 Lite	Size	CPU Model	Triple Rear Camera	Information not available
		6.5 inches, Aspect Ratio	Qualcomm Snapdragon 480 Plus	50 MP camera (*f*/1.8)+depth camera (*f*/2.4)+Macro camera (*f*/2.4)	
		20:9, Colour			
		16.7 million colours, Type	CPU Type		
		TFTLCD, Resolution	Octa-core processors		
		1,600*720, Gestures	CPU Dominant Frequency	Video Shooting	
		Multi-touch geatures, up to 10 touch points supported	2*A76*2.2GHz + 6*A55*1.9GHz	Support 1080 P video shooting	
			GPU		
				Focus Mode	
				Up to 8x digital zoom.	
				Image Resolution	
				Support up to 4,096 × 3,072 pixels	
			Adreno™ 619		
				*The actual image resolution may vary depending on the shooting mode.	
			Keyboard Type		
			Gestures, Three-key navigation, Navigation dock		
				Video Resolution	
				Support up to 1,920 × 1,080 pixels	
			Features		
				*The actual video resolution may vary depending on the shooting mode.	
			Face Recognition/One-Handed mode/App Lock/App Twin		
				Rear Flashlight	
				Support	
				Capture Mode	
				Rear Camera: Aperture, Night, Portrait(including beauty mode and bokeh), Pro, Panorama, HDR, Stickers, time-lapse photography, Super macro, High-res, dual-view, story, Capture smile	
	iPhone SE	Retina HD display	A15 Bionic chip	12MP Main camera	4K video recording at 24 fps, 25 fps, 30 fps or 60 fps
		4.7-inch (diagonal) widescreen LCD Multi-Touch display with IPS technology	6-core CPU with 2 performance and 4 efficiency cores	ƒ/1.8 aperture	
				Digital zoom up to 5x	1080 p HD video recording at 25 fps, 30 fps or 60 fps
			4-core GPU	Portrait mode with Depth Control	
					720 p HD video recording at 30 fps
			16-core Neural Engine		Extended dynamic range for video up to 30 fps
				Portrait Lighting with six effects	Optical image stabilisation for video
		1334 × 750-pixel resolution at 326 ppi			
					Digital zoom up to 3x
					LED true tone flash
				Optical image stabilisation	QuickTake video
					Slo-mo video support for 1080 p at 120 fps or 240 fps
		1,400:1 contrast ratio (typical)		True tone flash with slow sync	
					Time-lapse video with stabilisation
		True tone display			
					Night mode Time-lapse
					Cinematic video stabilisation (4K, 1080 p and 720 p)
		Wide colour display (P3)		Panorama (up to 63MP)	
		Haptic Touch		Sapphire crystal lens cover	
					Continuous autofocus video
		625 nits max brightness (typical)		Autofocus with Focus Pixels	
					Take 8MP still photos while recording 4K video
		Fingerprint-resistant oleophobic coating		Wide colour capture for photos and Live Photos	Playback zoom
					Video formats recorded: HEVC and H.264
		Display zoom		Deep Fusion	
					Stereo recording
		Reachability		Smart HDR 4	
				Photographic Styles	
				Advanced red-eye correction	
				Auto image stabilisation	
				Burst mode	
				Photo geotagging	
				Image formats captured: HEIF and JPEG	
	Lenovo S60	Capacitive touchscreen,	Qualcomm® Snapdragon™	Rear:	Information not available
		16M colors,	MSM8916 1.2 GHz 64-bit Quad Core	13MP auto-focus, LED Flash	
		5-point multitouch 5.0" HD (1280 × 720)			
		IPS display			
	Xiaomi Redmi 3	Information not available	Information not available	Information not available	Information not available
	Samsung Galaxy S4	Technology	CPU Type	Camera Resolution(Rear)	Video Codec
		FHD sAMOLED	Quad	CMOS, 13MP Camera Resolution(Front)	MPEG4, H.263, H.264, DivX, DivX3.11, VC-1, VP8, WMV7 / 8, Sorenson Spark, HEVC
		Colour Depth	CPU Speed		
		16M	1.9GHz	CMOS, 2MP Flash	
		Size		Power LED (1EA) Auto Focus Yes	
		5"			Video Resolution
		Resolution			Full HD (1080 p) Video Playback
		1,920 × 1,080			
					Video Frame rate
					30fps
					Audio Codec
					MP3, AAC, AAC+, eAAC+, AMR-NB / WB, OGG, FLAC, AC-3, apt-X (Bluetooth)
	Samsung Galaxy J5	Size (Main Display)	CPU Speed	Main Camera—Resolution	Information not available
		5.2” (131.8 mm)	1.6GHz		
		Resolution (Main Display)	CPU Type	CMOS 13.0 MP	
		720 × 1,280 (HD)	Octa-Core	Main Camera—F Number	
				F1.7	
		Technology (Main Display)		Main Camera—Auto Focus	
		Super AMOLED			
		Color Depth (Main Display)		Yes	
		16M		Front Camera—Resolution	
		S Pen Support			
		No		CMOS 13.0 MP	
				Front Camera—F Number	
				F1.9	
				Main Camera—Flash	
				Yes	
				Video Recording Resolution	
				FHD (1,920 × 1,080) @30fps	
	Lenovo Vibeshot	Capacitive touchscreen,	64-bit Qualcomm® Snapdragon™ 615 1.7 GHz	Rear:	Information not available
		16M colors, 5-point multitouch 5.0" (1,920 × 1,080) Full HD		16MP AF with true 16:9 BSI	
			Octa Core	sensor, tricolor flash, OIS,	
				IR sensor, 6P lens with blue glass	
		IPS display @ 440 ppi		filter and sapphire cover	
	Lenovo S750	Information not available	Information not available	Information not available	Information not available
	Huawei P10	Information not available	Information not available	Information not available	Information not available
	Samsung Galaxy A3	Size (Main Display)	CPU Speed	Main Camera—Resolution	Information not available
		4.7" (120.4 mm)	1.5GHz	CMOS 13.0 MP	
		Resolution (Main Display)	CPU Type	Main Camera—F Number	
		720 × 1,280 (HD)	Quad-Core	f/1.9	
				Main Camera—Auto Focus	
				Yes	
				Front Camera—Resolution	
				CMOS 5.0 MP	
		Technology (Main Display)		Front Camera—F Number	
		Super AMOLED		f/1.9	
		Color Depth (Main Display)		Main Camera—Flash	
		16M		Yes	
		S Pen Support		Video Recording Resolution	
		No			
				FHD (1,920 × 1,080) @30fps	
	iPhone 6S	Retina HD display with 3D Touch	A9 chip with 64-bit architecture	12-megapixel camera	4K video recording at 30 fps
			Embedded M9 motion coprocessor	Live Photos with stabilization	
					1080 p HD video recording at 30 fps or 60 fps
		4.7-inch (diagonal) widescreen LCD Multi-Touch display with IPS technology			720 p HD video recording at 30 fps
				Autofocus with Focus Pixels	
					Optical image stabilization for video (iPhone 6s Plus only)
				Optical image stabilization (iPhone 6s Plus only)	
		1,334-by-750-pixel resolution at 326 ppi			True tone flash
					Slo-mo video support for 1080 p at 120 fps and 720 p at 240 fps
				True tone flash	
				Panorama (up to 63 megapixels)	
					Time-lapse video with stabilization
		1,400:1 contrast ratio (typical)			
					Cinematic video stabilization (1080 p and 720 p)
				Auto HDR for photos	
				Exposure control	
					Continuous autofocus video
				Burst mode	Noise reduction
					Take 8-megapixel still photos while recording 4K video
		500 cd/m2 max brightness (typical)		Timer mode	
				ƒ/2.2 aperture	Playback zoom
				Five-element lens	3x digital zoom
				5x digital zoom	Face detection
				Hybrid IR filter	Video geotagging
				Backside illumination sensor	
		Full sRGB standard			
				Sapphire crystal lens cover	
		Dual-domain pixels for wide viewing angles		Auto image stabilization	
				Local tone mapping	
				Noise reduction	
				Face detection	
				Photo geotagging	
		Fingerprint-resistant oleophobic coating on front			
		Support for display of multiple languages and characters simultaneously			
		Display zoom			
		Reachability			
Nemcova et al.*,* (39)	Xiaomi Mi9	Information not available	Information not available	Information not available	Information not available
Scully et al.*,* (32)	Motorola Droid	Information not available	Information not available	Information not available	Information not available
Yan et al.*,* (36)	iPhone 6S	Retina HD display with 3D Touch	A9 chip with 64-bit architecture	12-megapixel camera	4K video recording at 30 fps
			Embedded M9 motion coprocessor	Live Photos with stabilization	
					1080 p HD video recording at 30 fps or 60 fps
		4.7-inch (diagonal) widescreen LCD Multi-Touch display with IPS technology			
					720 p HD video recording at 30 fps
				Autofocus with Focus Pixels	
					Optical image stabilization for video (iPhone 6s Plus only)
				Optical image stabilization (iPhone 6s Plus only)	
		1,334-by-750-pixel resolution at 326 ppi			True tone flash
					Slo-mo video support for 1080 p at 120 fps and 720 p at 240 fps
				True tone flash	
				Panorama (up to 63 megapixels)	
					Time-lapse video with stabilization
		1,400:1 contrast ratio (typical)			Cinematic video stabilization (1080 p and 720 p)
				Auto HDR for photos	
		500 cd/m^2^ max brightness (typical)		Exposure control	Continuous autofocus video
				Burst mode	
				Timer mode	Noise reduction
				ƒ/2.2 aperture	Take 8-megapixel still photos while recording 4K video
				Five-element lens	
				5x digital zoom	
				Hybrid IR filter	Playback zoom
				Backside illumination sensor	3x digital zoom
		Full sRGB standard			Face detection
				Sapphire crystal lens cover	
				Auto image stabilization	
		Dual-domain pixels for wide viewing angles		Local tone mapping	Video geotagging
				Noise reduction	
		Fingerprint-resistant oleophobic coating on front		Face detection	
				Photo geotagging	
		Support for display of multiple languages and characters simultaneously			
		Display zoom			
		Reachability			

### Criterion measurement characteristics

Criterion measurement characteristics are reported in Figure 2B and Table 5. 12-Lead ECG was used in two studies (20%) (11, 36), three studies (30%) used 5-lead (31, 32, 35), one study (10%) used 4-lead (37), two studies (20%) used 2-lead (33, 34), and two studies (20%) used 1-lead (38, 39). Six studies (60%) reported ECG electrode placement and sampling frequency (32, 34, 35, 37–39).

**Table 5:** Methodology of included studies.

Reference	Electrocardiogram (ECG) utilized	Electrode placement	ECG Processing information
Bánhalmi et al.*,* (37)	4-lead Cardiax PC-ECG device	Four electrodes connected to the four limbs of the participants (3 channel data)	∼500 Hz
Bolkhovsky, Scully and Chon (31)	5-lead ECG HP 78354A system	Not reported	Not reported
Drijkoningen et al.*,* (11)	12-lead ECG	Not reported	Not reported
Matsumura and Yamakoshi (33)	2-lead ECG	Not reported	Not reported
Matsumura et al.*,* (34)	2-lead ECG (Kanazawa University)	Spot electrode at the wrist, left leg and body earth.	All signals were sampled using an A/D converter at a rate of 1 kHz with a resolution of 16 bits, and stored digitally in a computer
Nam et al.*,* (35)	5-lead ECG HP 78354A system	Standard 5-lead configuration	1,000 Hz
Nemcova et al.*,* (38)	1-lead Bittium Faros 180 ECG	Recording of one bipolar lead of ECG signal from the chest	Sampling frequency of up to 1,000 Hz
Nemcova et al.*,* (39)	1-lead Bittium Faros 360 ECG	Electrodes attached to the chest according to device manual	1,000 Hz
Scully et al.*,* (32)	5-lead ECG HP 78354A system	Standard 5-lead configuration	400 Hz
Yan et al.*,* (36)	12-lead ECG (GE Series 2,000)	Not reported	Not reported

Hz, hertz*.*

### Experimental procedure characteristics

Environmental procedure characteristics are reported in Figure 2C and Table 6. Nine studies (90%) provided participant instructions (11, 31–38) and two studies (20%) had dietary restrictions (33, 34). Participant postures were stated in six studies (60%) (31, 33–35, 37, 39). Of these studies four (40%) were measured in seated posture (33–35, 37) and two studies (20%) were measured participants in two or more postures (31, 39). All studies (100%) reported measurement site (11, 31–39). Of these studies four (40%) were measured at the index finger (left) (32–35), two (20%) at the index finger (right) (11, 31), and four (40%) at the index finger (left or right not reported) (36–39). Two studies (20%) reported breathing pattern (32, 35) and participants were instructed to breathe at various metronome rates. Environmental conditions were reported in three studies (30%) (33, 34, 36) and not reported in the remaining seven (70%) (11, 31, 32, 35, 37–39). Stabilization period was reported in five studies (50%) (33–37). Of these studies one (10%) allowed participants 10 min for stabilization (33), two studies (20%) allowed participants 5 min (34, 36), one (10%) was permitted 20 s (37), and one (10%) was given an unspecified stabilization period (35). All other studies (50%) did not report stabilization period (11, 31, 32, 38, 39). Measurement duration was reported in all ten studies (100%) (11, 31–39). Finally, the number of attempts allowed per participant was reported in five studies (50%) (31–35). No study reported skin temperature and one study (38) measured contact pressure using the strain gauge array under the screen (3D Touch) but this was for the estimate of BP, and the actual force values were not reported.

**Table 6:** Methodology of included studies.

Reference	Participant instructions	Dietary control	Participant posture	Region of interest (right or left)	Breathing pattern	Environmental conditions	Stabilization period	Duration of measurement	Number of attempts
Bánhalmi et al.*,* (37)	Participants were instructed to sit in a relaxed position without talking during measurements	Not reported	Seated	Index finger (Not reported)	Not reported	Not reported	20 s ‘practice’ to locate rear facing camera with index finger.	5 min	Not reported
Bolkhovsky, Scully and Chon (31)	Participants were in a supine position and were instructed to place their right index finger on the camera lens of either the iPhone or Motorola smartphones. Participants were then instructed to sit up in the chair in the tilt position where measurements were repeated.	Not reported	Seated and supine	Index finger (right)	Not reported	Not reported	Not reported	iPhone: 2 min, and Motorola: 5 min	2 (1 measurement per posture)
Drijkoningen et al.*,* (11)	Asked to keep the smartphone in right hand during measurement.	Not reported	Not reported	Index finger (right)	Not reported	Not reported	Not reported	1 min	Not reported
Matsumura and Yamakoshi (33)	Participants were instructed to remain as still as possible	Prior to testing participants abstained from medication for 24 h, the consumption of food and caffeinated substances, and from intense physical activity for 2 h	Seated	Index finger (left)	Not reported	The experiment was conducted in a sound attenuated room, maintained at a temperature of 27–28 °C	10 min	BL: final 3 min of 10-minute stabilization	3 (one BL, on MA, and one MT)
								MA: 3-minutes	
								MT: 3-minutes	
								Measurements separated by 5 min rest interval.	
Matsumura et al.*,* (34)	Participants were instructed to sit quietly with the smartphone positioned in their left hand, placed on a cushion on their knee. They were instructed to grip the smartphone firmly, despite the existence of motion artifact	Participants were asked in advance to refrain from any medication from the previous day of laboratory testing and, for 2 h before laboratory testing, to avoid consumption of food and caffeine-containing substance, intense physical activity, and smoking.	Seated	Index finger (Not reported)	Not reported	Sound-attenuated experimental room, maintained at a temperature of 24–26°C	5 min	3 min (20 s sets, 10 s rest between sets)	2 sets of 3 x (20s + 10s)
Nam et al.*,* (35)	Participants were instructed to breathe at a metronome rate, in an upright seated position, whilst placing their index finger on the rear-facing camera lens of the smartphone, which was positioned on a table. The front facing camera recorded chest/abdominal movements.	Not reported	Seated	Index finger (left)	Instructed to breathe at a metronome rate whilst the front facing camera recorded chest/abdominal movements	Not reported	All subjects were acclimated to different metronome breathing rates	2 min	7 per subject
Nemcova et al.*,* (38)	The smartphone provided audio-visual feedback to the measured subject, indicating whether the placement of the smartphone was adequate to provide signals of sufficient quality. The participant adjusted the position of the smartphone based on the application feedback (particularly by the identification of quasi-periodic peaks/spikes seen in the signals). A flat signal or a signal with many peaks/spikes with the absence of quasi-periodicity represented a low-quality signal.	Not reported	Not reported	Index finger (Not reported)	Not reported	Not reported	Not reported	20 and 15 s (training and testing data, respectively)	Not reported
Nemcova et al.*,* (39)	Not reported	Not reported	Measurements 1, 2 & 3 seated. Measurement 4, walking/moving	Index finger (Not reported)	Not reported	Not reported	Not reported	30 s	Not reported
Scully et al.*,* (32)	Participants were instructed to place their left index finger over the camera lens, with the flash on, without pressing down with additional force	Not reported	Not reported	Index finger (left)	Participants inhaled with each beat of the metronome at 12, 18 and 24 beats per minute. Each metronome recording was 2-minutes in length.	Not reported	Not reported	2 min	1 spontaneous (single subject). 3 metronome rates
Yan et al.*,* (36)	Participants were instructed to not speak and remain still during measurements	Not reported	Not reported	Index finger (Not reported)	Not reported	The median backdrop and background light intensity during signal acquisition was 199 lux (IQR 127–249)	5 min	20 s	Not reported

### Primary outcome measure(s) and results of included studies

Seven studies (70%) reported mean and standard deviation for HR acquisition via criterion ECG (31–36, 38) and eight studies (80%) reported mean and standard deviation for HR acquisition via smartphone PPG measurement (31–36, 38, 39). Only one study (10%) reported the mean difference and its significance between criterion and index measurement (36) (Table 7).

**Table 7:** Primary outcome measure(s) and results of included studies. .

Reference	Device/conditions	*N*	ECG (Mean ± SD)	PPG (Mean ± SD)	Mean Diff.	P-Value
Bánhalmi et al.*,* (37)	iPhone 6	50	Not reported	Not reported	Not reported	Not reported
Bolkhovsky, Scully and Chon (31)	iPhone 4s supine:	9	70.8 ± 12.2	70.7 ± 12.1	Not reported	Not reported
	iPhone 4s tilt:	9	75.8 ± 12.0*	75.8 ± 11.9*		
	Droid supine:	13	71.9 ± 7.9	71.7 ± 7.9		
	Droid tilt:	13	77.4 ± 6.9*	77.1 ± 7.3*		
Drijkoningen et al.*,* (11)	Samsung Galaxy S4	28	Not reported	Not reported	Not reported	Not reported
Matsumura and Yamakoshi (33)	Rest	12	71.0 ± 9.6	71.2 ± 9.8	Not reported	Not reported
	MA	12	86.7 ± 14.7	86.8 ± 14.6		
	MT	12	75.1 ± 12.3	75.4 ± 12.1		
Matsumura et al.*,* (34)	HR-BL	12	69.8 ± 7.7	Red: 69.8 ± 7.7, Green: 70.0 ± 7.8, Blue: 69.9 ± 7.9	Not reported	Not reported
	HR-HMA	12	70.4 ± 8.2	Red: 70.2 ± 8.3, Green: 70.5 ± 8.3, Blue: 70.4 ± 8.6		
	HR-VMA	12	70.3 ± 9.3	Red: 70.3 ± 9.1, Green: 70.4 ± 9.2, Blue: 70.5 ± 9.2		
Nam et al.*,* (35)	HTC One M8 (All BR at rest)	11	74.9 ± 7.4	74.8 ± 8.0	Not reported	Not reported
Nemcova et al.*,* (38)	Training dataset	30	83.97	83.4	Not reported	Not reported
	Testing dataset (Lenovo Vibe S1)	10	71.3	71.8		
		10	70.7	69.3		
	Testing dataset (Various smartphones)					
Nemcova et al.*,* (39)	Xiaomi Mi9	12	Not reported	SWT -2nd Band (3.8–7.5 Hz): 119	Not reported	Not reported
				SWT -3rd Band (1.9–3.8 Hz): 117		
				SWT -4th Band (0.94–1.9 Hz): 96		
				SWT -5th Band (0.47–0.94 Hz): 54		
				SWT -6th Band (0.23–0.47 Hz): 30		
Scully et al.*,* (32)	Motorola Droid R	1	92.2 ± 5.3	92.3 ± 5.9	Not reported	Not reported
Yan et al.*,* (36)	iPhone 6S	40	73.46 ± 12.74	73.41 ± 12.60	−0.05 (1.03)	0.69

* Represents significant difference (*p* < 0.05) between supine and tilt position with paired samples *t*-test: BL, baseline; HMA, horizontal motion artifact; VMA, vertical motion artifact; SWT, stationary wavelet transforms; Hz, hertz; MA, mental arithmetic; MT, mirror tracing.

### Measures of validity

Correlations were reported in seven studies (70%) (11, 31, 34–38), Limits of agreement (LoA) (Bland-Altman method) were calculated in seven studies (70%) (11, 31, 33–37), ANOVA, Tukey *HSD* and geometric mean regression (GMR) were utilized concurrently in one study (10%) (34), Wilcoxon ranked sum test, were reported in two studies (20%) (36, 38), paired student *t* test were reported in one study (10%) (36), one study (10%) reported mean ± SD only (32) and one study (10%) utilized a non-numeric technical validation method of five expert reviewers (39). All studies (100%) (11, 31, 33–39) reported agreement, ranging from good to very strong and correlations ranging from *r *= .98 to 1, between HR-PPG and HR-ECG utilizing the methods outlined above (Table 8).

**Table 8:** Results for heart rate: correlations, measures of validity and summary of results.

Reference	Results	Measure of validity	Summary of result
Bánhalmi et al.*,* (37)	HR (b/min) results:	Pearson correlation, Lin. *m* and *b* represent the coefficients for the linear regression on HRV (PRV) with the corresponding mean error (err) (MSE), R^2^ is the coefficient of determination, and bias, SD, and BAR values are the results of the Bland-Altman analysis.	Significant very strong correlation between smartphone device/application and Cardiax PC-ECG device (*r* = 1, *P* = <0.001).
	Pearson correlation: 1		
	*P* value: <10^−23^		
	Lin. *m*: 1.00		
	Lin. *b*: −0.12		
	Lin. err (MSE): 0.011		
	Lin. *R^2^*: 1		
	Bias: 0.032		
	SD: 0.110		
	BAR: <0.001		
Bolkhovsky, Scully and Chon (31)	iPhone 4s supine: Pearson correlation: >.99	Pearson correlation and Bland-Altman method to calculate 95% LoA.	Very strong correlation between both smartphone devices and 5-lead ECG HP 78354A system in both postures (supine, tilt) (Sig. not reported).
	LoA: 0.29		
	iPhone 4s tilt: Pearson correlation:>.99		
	LoA: 0.29		
	Droid supine: Pearson correlation:.98		
	LoA: 3.20		
	Droid tilt: Pearson correlation: >.99		
	LoA: 1.40		
Drijkoningen et al.*,* (11)	Correlation:	Correlation coefficient and Bland-Altman ratio (BAR).	Significant very strong correlation between smartphone device/application and 12-lead ECG device (*r* = .98, *P* = <.001) (De Ridder et al.*,* 2018). BAR indicated no significant changes (results not reported).
	*R*^2^ (%): 95.7		
	*P*-Value: <.001		
Matsumura and Yamakoshi (33)	Correlation:	ANOVA, Tukey *HSD*, GMR [95% CI], and Bland-Altman method to calculate 95% LoA.	Very strong correlation (*r *= .999) between iPhysioMeter and 2-lead ECG measured by geometric mean regressions and *r *= .060 measured by Bland-Altman method (Sig. not reported).
	GMR = .999 [.9985, .9993],		
	BAP = .060 [−.131, .246]		
	Bias: −0.20		
	SD: 0.63		
	LoA: −1.43, 1.03		
Matsumura et al.*,* (34)	Red:	Mean (SD), Pearson correlation [95% CI], ANOVA, Tukey *HSD*, GMR [95% CI], and Bland-Altman method to calculate 95% LoA.	Very strong agreement for R, G and B channels measured with iPhone 4s (*r *= .9960, .9991 and.9975, respectively) in comparison with 2-lead ECG. For HR, the repeated-measures ANOVA did not reveal any significant main effects of measurement, *F*(3, 33) = 2.39, *p* = 0.119, *ε* = 0.63, *ηp*^2^ = 0.18, and condition, *F*(2, 22) = 0.25, *p* =thinsp;0.783, *ηp*^2^ = 0.02, and measurement × condition interaction, F(6, 66) = 0.76, p = 0.502, ε = 0.40, ηp^2^ = 0.06.
	GMR-R: 0.9960 [.9935, .9975]		
	BAP-R: 0.03 [−0.20, 0.26]		
	Bias: 0.10		
	SD: 0.74		
	LoA: −1.36, 1.56		
	Green:		
	GMR-G: 0.9991 [.9985, .9994]		
	BAP-G: −0.14 [−0.36, 0.10]		
	Bias: −0.12		
	SD: 0.36		
	LoA: −0.83, 0.58		
	Blue:		
	GMR-B: 0.9975 [.9961, .9985]		
	BAP-B: −0.29 [−0.48, −0.06]		
	Bias: −0.07		
	SD: 0.61		
	LoA: −1.27, 1.13		
Nam et al.*,* (35)	Bias: 0.12	Pearson correlation [95% CI], LoA, and Bland-Altman plot method to calculate 95% LoA.	Bland-Altman and correlation plots, both show good agreement with non-statistically significant bias, in HR between the green color band of the HTC camera and ECG measurements.
	LoA: −5.58, 5.52		
Nemcova et al.*,* (38)	Training dataset:	Wilcoxon rank-sum test, Pearson and Spearman correlation [95% CI].	Wilcoxon p-value is higher than *α* = 0.05 for both datasets. The null hypothesis (H0: there is no relationship between estimated and reference values) was rejected (*p* < 0.05) in all cases.
	Pearson R: 0.9844		
	Spearman P: 0.9796		
	Wilcoxon test: 0.8298		
	Pearson: 1.14E-22		
	Spearman: 4.71E-21		
	MAE: 1.3 bpm (1.61%)		
	MAX: 4 bpm		
	Testing dataset:		
	Pearson R: 0.9907		
	Spearman P: 0.9902		
	Wilcoxon test: 0.9136		
	Pearson: 4.95E-17		
	Spearman: 7.92E-17		
	MAE: 1.4 bpm (1.89%)		
	*P* < 0.05 [all values]		
Nemcova et al.*,* (39)	Total 48 signals.	Technical validation of waveforms from five expert annotators.	31 of 48 signals were regarded “good quality” and had a HR error equal or lower than 5 bpm for each expert.
	Of 48 signals, 31 were regarded “good quality” according to all annotators.		
Scully et al.*,* (32)	The mean ± SD was 92.2 ± 5.3 bpm for HR-ECG and 92.3 ± 5.9 bpm for HR-GREEN.	Mean (SD) Diff.	Authors confirmed the accuracy of HR-GREEN vs. HR-ECG (Sig. not reported).
Yan et al.*,* (36)	Pearson R:.997 (*P *< .001)	Mean (SD) Diff., Pearson R, Wilcoxon rank-sum test, Paired student *t* test, Bland-Altman plots.	Significant very strong correlation between smartphone device/application and 12-lead ECG device (*r* = .99, *P* = <.001).
	*R*^2^ (%): 99.3		
	Wilcoxon test: 0.53		
	Paired student *t* test: 0.69		
	Bias: 0.046		
	LoA: −1.98, 2.07		

## Discussion

### Principle findings

This scoping review provided an overview of existing literature regarding the acquisition and validity of HR-PPG, in healthy subjects at rest utilizing smartphone devices, with the aim of facilitating improvements in future research and clinical practice. In relation to our objective of assessing the validity of HR-PPG acquisition from PPG measurement utilizing contact-based smartphone devices, this review highlighted several methodological and reporting discrepancies between studies which can lead to different results that do not reflect outcome of comparison (22). As there is currently no consensus on what metric should be used to establish the validity of smartphone-based PPG or under what conditions, the reviewed research appears to have utilized an exploratory approach. However, with the rapid development in technology and an improved understanding of this research area, we have highlighted key considerations for reporting contact-based PPG RHR acquisition with smartphones (Table 1).

### Target population considerations

With regards to the general study information reported (Table 2) results revealed only one study (10%) (37) met the suggested guidelines for validating heart rate devices (albeit wearables) suggested by Mühlen et al. (40). Overall reporting was poor with small and unjustified sample sizes, and few studies adequately reported sex, skin color, or age of participants. An expert consensus suggested that studies validating HR-PPG should determine sample size based on an expected mean absolute difference, expected SD of differences and a pre-defined clinical maximum difference needed to obtain a power of 80% or 90% to assess agreement with sufficient precision (41). If no *a priori* level of “in agreement” is specified a sample size of 45 is recommended (42). However, all but one study had a sample size of *n* < 45, and therefore results could be under powered (43). We suggest sample sizes should be carefully calculated during study design utilising current guidelines (43) and these calculations should be presented in the methods section.

Comorbidities were poorly reported, particularly those that might affect pulse rate or amplitude, such as arterial stiffening or conditions affecting cardiac electrophysiology (33, 44). At a minimum, studies should report either that participants were free from such health conditions, or clearly state their health conditions, if their aim is to validate HR-PPG in a particular population.

Additionally, there was inadequate reporting of participant skin color within this review. Felix von Luschan chromatic scale (VLCS) (range 1–36) was utilized, which is a validated method of skin color evaluation (45). Skin color is an important consideration during PPG acquisition as skin tone may affect the accuracy of measurements (40). However, a recent systematic review of wrist-word devices, which utilize reflective PPG, stated evidence is inconclusive possibly due to small sample sizes and the requirement for a more objective way of identifying participants' skin tone (46). Nevertheless, authors suggested HR-PPG detection may be less accurate in darker skin tones (46). Since the papers in this review failed to adequately report skin tone this cannot be corroborated with regards to camera-based methods and although Yan *at al.* (36) measured skin tone (Table 2), participants fell within the mid-range of the skin tone spectrum (range 19–25), with one representing light skin and 36 representing dark skin (45). Consequently, it is not clear if participants' skin tone influenced the results of the studies in the present review. Therefore, human factors such as skin color should be recorded (47) and appropriate light wavelength should be selected (48). Moreover, it is evident that more research is required investigating the effect of darker skin tones on signal quality.

### Index measurement considerations

Interestingly, the majority of studies reported the use of a single smartphone device (11, 32–37, 39). This of course maximizes internal validity of each study, but does somewhat hamper ecological validity and generalizability, given the vast options in terms of smartphones at the time of writing. Additionally, since a major advantage of mHealth technologies are their reach, it is advisable to assess the index measurements validity cross-platform, at a minimum of one phone from each. Moreover, the most recent article was Nemcova et al. published in 2021 (28), suggesting future measurements could improve through the utilization of newer technology (14).

As highlighted in the results, heterogeneity existed between smartphone model and application utilized and although authors reported the name of the smartphone application, zero studies reported the specific programming code utilized for beat detection. This could be due to financial, security and/or privacy reasons, as some applications were commercially available. This makes direct comparisons between apps and devices difficult as there is no guarantee two apps used the same code. Additionally, around half of the studies stated the application utilized was developed specifically for the intended research, therefore the algorithm could have been described or the code made available. Consequently, validation of specific algorithms within this review was not possible, this could be feasible in future if algorithms and build versions were explicit (49). Moreover, it is difficult to extrapolate these data to the real world without testing the efficacy of those applications outside stringent conditions of a laboratory. Identification of certain smartphones or applications which produce better PPG signals could lead to improvements in HR measures (23). However, this is difficult as there is currently no consensus on what metric should be used to establish the validity of smartphone-based PPG or under what conditions, therefore protocols vary dramatically. Identifying optimal device(s) and application(s) is difficult. Therefore, we present a checklist (Table 1) to facilitate superior acquisition of HR-PPG via smartphone devices.

Although there has been a considerable increase in the number of mobile apps, many have been designed without regulation regarding development, risk mitigation, and quality control. Therefore, we advise future developers to adhere to the guidelines proposed by Llorens-Vernet and Miro (50), which consist of 36 important criteria and outline standards for mobile health-related applications. These criteria are grouped into eight categories including usability, privacy, security, appropriateness and suitability, transparency and content, safety, technical support and updates, and technology.

Most studies reported which camera recorded smartphone PPG measurements (32–39) of which the rear-facing camera was utilized for all with torch (flash) turned on. However, recent research investigating rear- vs. front-facing PPG smartphone measurement revealed the front-facing camera to be more advantageous when considering greater control over the emitted light and finger detection. It is possible that previous research has not utilized this method as smartphone devices with front-facing camera capabilities are a newer technology that is still under development (14). However, regardless of the camera selected it is advisable to state this as camera selection clearly influences PPG signal quality.

Over half of the studies reported camera resolution (32–35, 37, 39). However, it was not clear if the reported resolution was referring to the smartphone cameras hardware settings or if the resolution was selected through the applications capture settings. Raposo et al.*,* (14) suggest resolution should be set to its minimum value to reduce computational load. Moreover, implementation of interpolation techniques can be used to increase fiducial point detection through improvements in temporal resolution (51). This could influence device selection as future research could utilize devices with theoretically suboptimal resolution. For example, a device that, without adjustment of capture resolution would have high computational load, yet have other PPG performance advantages, we could then manually determine the resolution to the desired level within capture settings (i.e., reducing the capture resolution within the app), potentially improving PPG signal quality, and reducing computational load. For this reason, it is important to report what the resolution is and how it was acquired since newer devices often provide multiple rear-facing lenses, of which some have “slow-motion” technology, providing potentially enhanced sampling rate capabilities.

Smartphone sampling rate was reported in most studies (11, 31–35, 37–39). Sampling rate can be as high as 1,000 Hz for medical equipment (52) however, for most smartphone cameras, it is typically less than 30 Hz (53), which can result in inaccurate waveform analysis (54). As outlined in our results, sampling rate was generally 20–30 Hz. For context, the latest smartphone model in the reviewed studies was the iPhone 6s (released 2015), which has a sampling rate of 30, 60 or 240 Hz, depending on the resolution settings during recording. Implications of inappropriate sampling frequency selection could result in inaccurate waveform analysis (54) and HR-PPG determination. Beres and Hejjel (51) investigated the minimum sampling frequency requirements for HR-PPG parameters in healthy individuals and concluded a minimum of 5 Hz is sufficient without interpolation, for pulse rate determination. However, although lower sampling frequencies minimize the computational load and, as a result, the power consumption consequently extending battery life (51), they can also deteriorate the accuracy of fiducial point detection in HR-PPG and/or HR-ECG, decreasing signal accuracy. Moreover, applications intending on measuring other parameters, for example those related to HRV, would require higher sampling rates with possible interpolation (51). As sampling rate is largely determined by smartphone make/model, we advise future research to utilise devices with higher sampling rate capabilities and/or implement interpolation techniques. When designing an application, it is important to consider the parameter being measured (higher sampling rates required for HRV in comparison to HR analysis) and the target demographic, as applications that are compatible with newer and older smartphone models could provide for broader scope, especially for those in low- and middle-income countries (LMIC) that may not have access to adequate healthcare.

As various wavelengths interact differently with blood and tissues (55), important consideration must be had with regards to wavelength selection (56) (i.e., red, green or blue colour channels). Emerging research suggests green wavelengths demonstrated stronger cardiac pulse signals in comparison with red or blue bands during remote PPG imaging (37). However, this was demonstrated in wrist-worn devices and more research is required in smartphone-derived PPG. Finally, improvements in pulse signal could be attained through optimization of the pixel averaging region (32), whereby the video area closest to the light source is analysed increasing the overall gain of the signal and therefore improving signal quality (14).

### Experimental procedure considerations

Firstly, when describing the experimental procedure, studies described the technical computer science methods well. However, their relationship to physiology (i.e., what variable they are measuring and the relationship between the signal capture and the underpinning physiology) was not described in as much detail. Nearly all studies provided participant instructions (11, 31–38), however, some study designs were hard to follow and not enough detail was provided to allow accurate replication. Studies that provided sufficient detail utilized schematic diagrams and detailed subsections within the methods as to index and criterion measurements, experimental procedure, and participant instructions.

Over half the studies reported participant postures with the seated posture being the most frequently utilized measurement position. Postural changes can result in deviations in cardiovascular measurements, such as HR (57, 58). Therefore, participant measurement posture should be reported when describing the experimental procedure. In addition to measurement posture, measurement site is also an important consideration. Hartmann et al. (59) investigated the effect of measurement site on HR-PPG waveform characteristics utilizing a reflective PPG sensor with a peak wavelength of 880 nm, comparable with reflective wavelengths utilized in smartphone devices that utilize an infrared light wavelength (880–940 nm) (6). Authors determined that under normal and deep breathing conditions the finger produced the most analyzable waveforms (95% and 86% analyzable, respectively) in terms of mean amplitude, pulse peak time (Tp), dicrotic notch time (Tn), and the reflection index (RI) (all *p* < 0.001), which could be due to higher sensitivity to volumetric fluctuations in the cutaneous vascular walls of the finger compared with other measurement sites (59).

The application of pressure at the measurement site is something to be considered, as this is the fundamental of blood pressure measurement (i.e., an increase in pressure eventually results in occlusion). Variations in contact pressure can result in changes in several waveform characteristics (60). Increased contact pressure decreases the optical path length through the tissue, increasing AC amplitude. AC amplitude reaches its maximum when transmural pressure, defined as the difference between intraarterial pressure on the vessel wall and contact pressure, reach zero (61, 62). Additional pressure beyond this begins to occlude the vessel reducing amplitude until no signal is visible. Conversely, contact pressure applied too softly increases the optical path length through the tissue, decreasing AC amplitude. Considering this, applying enough pressure to create conditions where transmural pressure is zero could be beneficial for RHR determination, as this could make peaks more easily identifiable. While this paragraph briefly outlines the underlying physiology and AC amplitude changes form varying contact pressures, from a technical standpoint, Apple stopped incorporating the strain gauge array under the screen (3D Touch) from ∼2017 onwards. Therefore, no force measures can be obtained directly from the device. For this reason, our in-house pilot testing has suggested that providing the app user with the real-time PPG signal (i.e., visual feedback) can enhance the quality of the PPG signal. This approach has been previously conducted by Nemcova et al. (38) who reported they provided app feedback (visual peaks presented on the smartphone display) to enhance signal quality during measurement conditions. These authors stated that quality was evaluated visually by the users; quasi-periodic peaks/spikes must be seen in the signals. A flat signal or a signal with many peaks/spikes with the absence of quasi-periodicity represents a low-quality signal. The user should iteratively change the position of the smartphone according to the feedback of the application. Therefore, applying contact pressure which allows a signal which displays key pulse wave fiducial points, that has many quasi-periodic peaks would be considered ideal.

Previous research stated environmental conditions such as ambient light or motion can influence HR measurement (49, 63). In addition, careful consideration of the environmental temperature has the benefit of reducing possible HR-ECG and HR-PPG noise due to shivering (64). Of course, these environmental conditions ultimately influence participant temperature, and temperature of the measurement site (i.e., skin temperature). However, no study included in this review reported skin temperature. From a technical standpoint, the device temperature sensors are only designed for management of the CPU and battery, so measurement of environmental or skin temperature is beyond the scope of those sensors. Thus, skin temperature reporting would require an additional device such as a skin thermometer. From our in-house pilot testing, we have observed that having cold hands can reduce the quality of the PPG signal (by “quality” we mean a signal which displays key pulse wave fiducial points, that has many quasi-periodic peaks would be considered ideal). This in-house pilot testing in our lab is supported by previous work suggesting that both increased and decreased skin temperature can alter the increased PPG amplitude and total signal, PPG waveform amplitude, and PPT (60, 65–68). Research suggests ambient light may also affect light sensitive diodes; however, the size of the effect is currently unknown (40). Allen (47) suggests correct positioning of the device and the use of light modulation filtering can reduce ambient light interference.

We identified HR-ECG and HR-PPG were generally recorded simultaneously for short durations (<3 min), which is acceptable. Nemcova et al. (39) suggest ultrashort- (< 5 min) and short-term (∼ 5 min) measurements have several advantages over longer term measurements, including minimal risk of data loss during measurement, subject comfort (including flash/torch burn risk) and reduced computational demands that influence battery capacity and memory. Definitions of short- and ultra-short vary depending on the intended research, 10 s duration is commonly cited as the most appropriate duration within the literature, for HR-PPG acquisition. However, although all studies in the current review reported measurement duration (Table 6), no study compared the effect of increased or reduced measurement duration on signal quality. Therefore, the impact of measurement duration in the present review is unclear.

The time taken for a pulse wave to travel along a fixed arterial length is considered the pulse transit time (PTT). When that pulse arrives, known as pulse arrival time (PAT), it is represented by a peak in the HR-PPG signal, however due to the PTT, there is misalignment, or “time lag”, between the R wave of the HR-ECG signal and the HR-PPG peak (69). A recent review of open-source beat detection algorithms describes a method of time alignment where HR-ECG and HR-PPG derived beats within the range of <150 ms were determined to be correctly identified. The time lag between beats was manipulated by offsetting the beats in increments of 20 ms. The time lag that resulted in the most correctly identified beats (the most HR-ECG and HR-PPG beats within the range of <150 ms) was considered the “true lag” (70). Time alignment allows for direct beat comparison and ensures that not only are the same time frames are being analysed but also the same beats, improving validity assessment. However, only Bolkhovsky et al. (31) explicitly stated that HR-ECG and HR-PPG were aligned during post-recording data analysis.

Finally, the number of attempts allowed per participant was inadequately reported (Table 6). Holmes et al. (71) suggest number of attempts should be limited to three as additional measurements would counteract the advantages of ultrashort-term measures outlined above. We argue that there is a compromise to be made between end-user burden/acceptability and reliability/precision. Whilst it is likely that more trials per participant will increase the chances of acquiring a good signal and therefore improve validity, the more trials a user completes the greater the data entry burden (72), which could reduce usability and adherence.

### Primary outcome and statistical measures of validity considerations

Results of this scoping review highlight the agreement between HR-PPG and HR-ECG (Table 8). In this scoping review CIs, LoA, or bias [from which LoA can be derived (LoA = bias ± 1.96 SD)] were not reported in all studies. Yet Mühlen et al. (40) state 95% confidence intervals (CIs) and LoAs should be provided for between-device comparisons. Interestingly, given the large number of studies reporting correlation coefficients, zero papers defined guidelines utilized to determine strength of coefficients (73, 74). We conducted *post hoc* interpretation and six articles (11, 31, 34, 36–38) exceeded the minimum requirements for “high” or “strong” correlation using previously reported guidelines (73, 74). However, it was unclear whether these studies examined mean HR agreement, rather than time alignment and beat to beat agreement.

Nam et al. (35) stated PPG measured from the green wavelength (HR-Green) demonstrated “good agreement” (Table 8) in comparison with HR-ECG, however, neither the coefficient itself, nor the criteria for this qualitative assessment was provided. In summary, statistical interpretation could be improved in future research, utilizing the Bland-Altman method (75) for testing agreement between HR-ECG and HR-PPG, rather than relationship between the two (as agreement and relationship are different concepts). We also propose greater transparency in statistical reporting, including precise coefficients, *a priori* thresholds for interpretation (i.e., “poor”, “good”, “very good”) etc.

### Effects of mobile platform (iOS and android)

It is worth noting that there are technical and practical issues related to the platform (iOS *vs*. Android) used to collect PPG data. A common approach is splitting the captured image into its primary colour components since red or green channels often provide a better signal. This technique largely hinges on the sensor's colour sensitivity and its colour filter array (CFA) precision. Given the stricter manufacturing control, Apple's iOS devices have a more uniform sensor technology and will likely offer a consistent baseline for PPG measurements between devices. The wider range of Android manufacturers means devices will use sensors from different producers, resulting in a broader range of sensor metrics between devices. The distinct approaches to sensor integration and image processing algorithms may further compound these differences. Apple's control over hardware and software typically results in predictable sensor performance. In contrast, Android devices might exhibit significant variability in sensor behaviour, potentially impacting homogeneity of PPG measurements across devices.

While these differences mean different phones may have different magnitudes of sensor values, the degree to which this impacts peak detection or other variables (such as frequency domain HRV) has yet to be widely investigated. More broadly, both platforms offer frame rates that enable sampling at 30 Hz. While this is suitable for peak detection, more nuanced analyses, such as wave morphology and feature identification, are challenging at this frame rate. Both manufacturers have started to include higher framerate video capture, such as “slow motion” modes with frame rates between 120 and 240 Hz. While these modes may reveal more significant detail in the collected wave data, the validity and reliability of this higher framerate regarding critical variables (e.g., the consistency of the period between frames) is not known.

There are other more practical issues regarding using phones for widescale HR monitoring. While not a significant feature of this review, in our testing (our unpublished observation) we have found that the flash on some Android phones gets uncomfortably hot when used in bulb mode (necessary to generate the PPG data). Similarly, the trend for integrating more lenses into the phone has, in some cases, moved the lenses further from the flash, resulting in less consistent lighting across the tissue in contact with the lens (our unpublished observation).

## Conclusions and practical recommendations

To ensure validity and comparability with previous research, we have proposed a framework for optimal reporting (Table 1). This was based on the “Towards Intelligent Health and Well-Being: Network of Physical Activity Assessment” (INTERLIVE) best-practice recommendations (40). We took the INTERLIVE statement for wearable devices and adapted it for phone camera-based PPG. The validation process should consider six domains: the target population, criterion measure, index measure, testing conditions, data processing and the statistical analysis (40). Adherence to the checklist will result in superior acquisition of HR-PPG via smartphone devices, facilitating improvements in research and clinical practice. Future research could investigate validity with consideration towards effective approaches that transfer these methods from laboratory conditions into the “real-world”, in both healthy and clinical populations.

## Data Availability

The original contributions presented in the study are included in the article/Supplementary Material, further inquiries can be directed to the corresponding author.
